# Referral patterns and patient characteristics in pediatric feeding disorder care at a multidisciplinary outpatient clinic

**DOI:** 10.1002/jpr3.70237

**Published:** 2026-09-03

**Authors:** Edward Tannenbaum, Riad Rahhal, Linda Cooper‐Brown, Willow Cover, Adam Vaske, Dina Al‐Zubeidi

**Affiliations:** ^1^ Carver College of Medicine University of Iowa Iowa City Iowa USA; ^2^ Department of Pediatrics Gastroenterology, College of Medicine at Peoria University of Illinois Champaign Illinois USA; ^3^ Stead Family Department of Pediatrics‐Psychology University of Iowa Iowa City Iowa USA; ^4^ Stead Family Department of Pediatrics‐Gastroenterology University of Iowa Iowa City Iowa USA

**Keywords:** enteral nutrition, feeding tube, oral aversion

## Abstract

**Objectives:**

Pediatric feeding disorders (PFDs) are increasingly prevalent in the United States. Limited research has examined referral characteristics for outpatient feeding programs. This study analyzes patient referral characteristics based on referral patterns, patient demographics, and treatment interventions.

**Methods:**

A retrospective cohort study was conducted of pediatric patients evaluated in a multidisciplinary outpatient feeding clinic at a tertiary pediatric academic institution from 2021 to 2024.

**Results:**

We found a significant difference in the rate of referrals to our pediatric feeding program (*p* < 0.001), with primary care physician (PCP) referrals as the most common. There were significant differences in the rates of comorbidities (*p* = 0.004) and tube feeding (*p* < 0.001) among the referral sources, with specialist referrals associated with the highest rates and PCP referrals associated with the lowest rates. Oral aversion was common when a feeding tube was present, regardless of tube type.

**Conclusions:**

Given the high rate of referrals from PCPs, there is a significant opportunity to improve resource utilization and focus efforts on feeding disorders education for referring providers. In contrast, an accelerated pathway may need to be considered for patient referrals from specialists and neonatology due to higher comorbidities and the need for feeding tubes in this referral group. In the feeding tube category, our study also suggests that there may be no difference in the rates of aversive behaviors between different types of tubes used for enteral feeding; this may help guide provider/parent decision‐making during enteral access selection.

## Abstract (highlights)

**What Is Known:**

- Pediatric feeding disorders are common and best managed through a multidisciplinary approach.
- Limited literature suggests that primary care physicians may be the highest referral source to pediatric feeding programs.

**What Is New:**

- Specialist referrals involved patients with higher rates of comorbidities and tube feeding compared with primary care referrals.
- Differences among referral sources suggest opportunities to improve resource utilization through targeted educational initiatives.
- Expedited access pathways may help prioritize higher‐complexity patients.
- Oral aversion was commonly associated with feeding tube use regardless of tube type but was less prevalent among children using nasogastric tubes.

## 1 INTRODUCTION

Pediatric feeding disorders (PFDs) are increasingly common, with a prevalence in the United States between 1 in 23 and 1 in 37 children under the age of 5.^1^ One proposed framework defines PFD as an oral intake impairment that is inappropriate for age and associated with nutritional, feeding skill, medical, and/or psychosocial dysfunction.^2^ Timely recognition and management are essential, as untreated PFD can lead to growth faltering, developmental concerns, and reduced quality of life.^3^

Effective assessment and treatment of PFD often require a multidisciplinary approach due to the varied etiologies.^3^ Inpatient programs can be costly, and families often must relocate to access care.^4^ There has been limited investigation into the referral patterns and effectiveness of the outpatient treatment model.^5^, ^6^, ^7^ One study characterized the referral population at a pediatric outpatient feeding clinic, identifying poor nutrition, food refusals, and poor weight gain as the most common reasons for referral.^7^ Most referrals came from primary care physicians (PCPs), followed by rehabilitation specialists and self‐referrals.^7^

Further evaluating referral sources could help optimize resource use and guide targeted provider education. We hypothesized that PCPs would have the highest referral rates, with neonatology being the highest specialty referral. In addition, we theorized that specialists' referrals would be associated with patients with lower weight *z* scores as well as higher rates of comorbidities, esophagogastroduodenoscopies (EGDs), feeding tube use, intensive feeding therapy, and appetite stimulation medication, compared to PCP referrals. We further reviewed patients with oral aversion and its association with the type of feeding tube.

## 2 METHODS

### 2.1 Ethics statement

This study was approved by the University of Iowa Institutional Review Board.

### 2.2 Study design

We conducted a retrospective cohort study of patients followed at a large children's hospital pediatric feeding clinic from April 2021 to April 2024. Patients were identified through a query of the electronic health record utilizing Epic's SlicerDicer tool. We queried these data for new visits to the Pediatric Feeding Clinic with the following criteria: age greater than 6 months and less than 18 years and initiation of treatment with the multidisciplinary team (physician, psychologist, speech‐language pathologist, and dietician). Our clinic defines PFD as a child's inability or refusal to eat or drink sufficient quantities to maintain nutritional status. Eligibility criteria for referral include behavioral feeding difficulties, aversion to specific textures, difficulty chewing, developmental delays, severe gastroesophageal reflux, poor weight gain, mealtime challenges, picky eating, poor appetite, constipation, management of tube feeding, and a history of repaired anatomic abnormalities. Patients were excluded if they were not evaluated by the full multidisciplinary team at the initial visit.

De‐identified data were collected from electronic medical records, including patient demographics, anthropometrics, diagnoses and referral sources based on the initial visit. Growth charts were utilized for weight *z* scores and weight‐for‐length z‐scores. The use of EGD or tube feeding was documented. The history of feeding tube use was further categorized as use of a nasogastric (NG) tube only, use of any other type of feeding tube, and no use of any feeding tube. The use of appetite‐stimulating medication or intensive feeding therapy was recorded. Intensive feeding therapy at our facility often involved multiple outpatient sessions in a week, over weeks or months, working with the patient to address learned behaviors, sensory challenges, and oral motor issues (such as chewing). Parents and caregivers participated in coaching and training to implement treatment strategies at home. Psychology was the primary discipline working with the child during intensive feeding sessions, but other disciplines also participated as needed (e.g., speech‐language pathologist).

Analysis of interventions, including EGD, appetite‐stimulating medication use, tube feeding, and intensive feeding therapy, was performed based on referral sources. Referral sources were categorized as PCP, neonatology, pediatric gastrointestinal physician (PED GI), or other specialties. Neonatology referrals were made directly through the high‐risk neonatal intensive care unit follow‐up clinic. Other specialties included cardiology, neurology, orthopedics, otolaryngology, and hematology/oncology. The referral sources were further categorized as “internal” if they were from a provider within our institution or “external” if they were referred outside of our institution.

### 2.3 Outcomes

The primary outcome was to assess differences in patient characteristics based on referral patterns. Secondary goals included evaluation of tube feeding and oral aversion.

### 2.4 Statistical analysis

Demographic and clinical variables were summarized using counts and percentages for categorical measures. Continuous variables that were non‐normal in distribution and had a Shapiro–Wilk *p*‐value > 0.05 were summarized using medians and interquartile ranges, while variables that were approximately normal in distribution were summarized using means and standard deviations. To test for differences in these variables between referral sources or tube types, Fisher's exact test was used for categorical variables, one‐way analysis of variance was used for normally distributed continuous variables, and the Kruskal–Wallis rank sum test was used for non‐normally distributed variables. When comparing demographic and clinical measures across dichotomous variables, Fisher's exact test was still used for categorical variables, while Welch's two‐sample *t* tests and Wilcoxon rank‐sum tests were used for normally and non‐normally distributed variables, respectively.

A *χ* ^2^ test was used to examine whether the four referral source categories were equally prevalent, and binomial tests were used to make pairwise comparisons between the different referral sources. R version 4.5.0 was used for all analyses, and *p* values < 0.05 were considered statistically significant.

## 3 RESULTS

### 3.1 Demographics

In the aggregate, 148 children were evaluated in the multidisciplinary pediatric feeding clinic for an initial visit. The median age at referral was 14 months (Q1, Q3: 10, 34). Approximately 46% (68/148) were female. At referral, the average weight *z* score was –1.10 (*σ* = 1.53). The average referral weight‐for‐length *z* score and median body mass index (BMI) were –0.38 (*σ* = 1.45) and 16.02 (Q1, Q3: 14.80, 17.66), respectively.

Approximately 47% (68/146) and 39% (56/145) of patients had at least four comorbidities and at least one gastrointestinal (GI) comorbidity, respectively. Some of the most common comorbidities included constipation, gastroesophageal reflux disease, developmental delay, bronchopulmonary dysplasia, trisomy 21, and autism spectrum disorder. The percentage of patients with a history of tube feeding was 64% (90/141). Twenty percent (29/144) of patients had undergone an evaluation with EGD. Approximately 17% (24/143) were treated with appetite‐stimulating medication and 10% (15/143) received intensive feeding therapy (Table 1).

**Table 1:** Referring provider comparison of baseline characteristics of patients seen in the multidisciplinary pediatric feeding clinic.

Characteristic	*N*	Overall	All other subspecialties	Neonatology	PCP	Ped GI	*p* value
Referral age, median (Q1, Q3)	148	14 (10, 34)	18 (15, 37)	9 (7, 13)	19 (11, 58)	14 (10, 34)	**<0.001**
Female, *n* (%)	148	68 (46%)	10 (40%)	14 (61%)	25 (42%)	19 (48%)	0.4
Weight *z* score at referral, mean (*σ*)	142	−1.10 (1.53)	−0.63 (1.45)	−1.76 (1.39)	−0.96 (1.49)	−1.19 (1.62)	0.068
Weight‐for‐length *z* score at referral, mean (*σ*)	100	−0.38 (1.45)	−0.20 (1.83)	−0.27 (1.47)	−0.39 (1.43)	−0.54 (1.27)	0.9
BMI at referral, median (Q1, Q3)	141	16.02 (14.80, 17.66)	15.98 (14.41, 18.25)	17.37 (15.63, 18.01)	15.96 (14.78, 17.62)	15.73 (15.20, 16.91)	0.4
≥1 GI comorbidity, *n* (%)	145	56 (39%)	6 (24%)	9 (39%)	19 (33%)	22 (56%)	**0.043**
≥4 comorbidities, *n* (%)	146	68 (47%)	13 (52%)	17 (74%)	18 (31%)	20 (50%)	**0.004**
History of tube feeding, *n* (%)	141	90 (64%)	15 (63%)	21 (91%)	25 (45%)	29 (74%)	**<0.001**
Use of endoscopy, *n* (%)	144	29 (20%)	5 (20%)	2 (8.7%)	10 (17%)	12 (32%)	0.2
Appetite‐stimulating medication use, *n* (%)	143	24 (17%)	4 (16%)	2 (8.7%)	11 (19%)	7 (18%)	0.7
Use of intensive feeding therapy, *n* (%)	143	15 (10%)	3 (12%)	2 (8.7%)	5 (8.8%)	5 (13%)	0.9
External, *n* (%)	148	41 (28%)	3 (7.3%)	0 (0%)	37 (90%)	1 (2.4%)	**<0.001**
Internal, *n* (%)	148	107 (72%)	22 (21%)	23 (21%)	23 (21%)	39 (36%)	0.057

*Note*: Bold values indicate statistically significant.

Abbreviations: BMI, body mass index; PCP, primary care physician; Ped GI, pediatric gastrointestinal physician.

### 3.2 Referral sources and their characteristics

**Figure 1:**
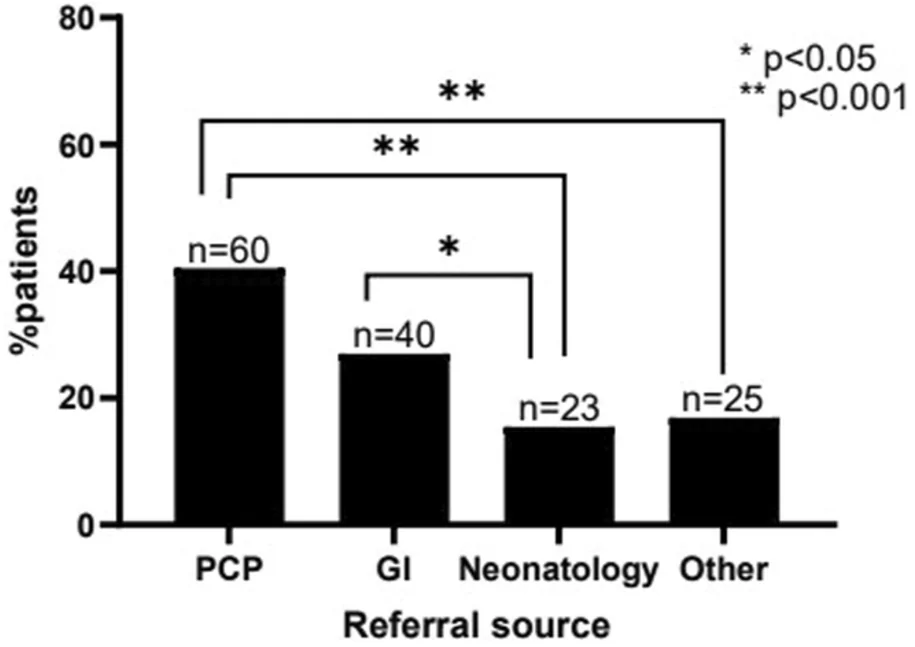
Pediatric feeding program referral rates by source. GI, pediatric gastrointestinal physician; PCP, primary care physician.

In comparing the four referral sources, there was a significant difference in the rate of referrals among the different referring groups (*p *< 0.001). The most common referral source to the pediatric feeding program was from PCPs, followed by PED GI. There were 60 (40.5%) patients referred from PCPs and 40 (27.0%) patients referred from PED GI. Twenty‐five (16.9%) patients and 23 (15.5%) patients were referred from all other subspecialties and neonatology, respectively. Specific pairwise differences were also found between all other subspecialties and PCPs (*p* < 0.001), neonatology and PCPs (*p* < 0.001), as well as neonatology and PED GI (*p* = 0.043) (Figure 1).

The prevalence of four or more comorbidities differed significantly across referral sources, with neonatology referrals having the highest percentage of 74% and PCP referrals having the lowest percentage of 31% (Table 1).

A significant difference was noted in the rate of tube feeding use among the four referral sources. Neonatology referrals had the highest rate at 91%, and PCP referrals had the lowest rate at 45% (Table 1).

When comparing referrals from neonatology and PCPs, several differences in patient characteristics were found. Age at referral was younger at 9 months old in neonatology referrals compared to 19 months old in PCP referrals (*p* < 0.001). The weight *z* score at referral was lower at –1.76 (*σ* = 1.39) in the neonatology group compared to –0.96 (*σ* = 1.49) in the PCP group (*p* = 0.030). In addition, the rate of four or more comorbidities was significantly higher in patients referred by neonatology at 74% compared to patients referred by PCPs at 31% (*p* < 0.001). Similarly, the rate of tube feeding was significantly higher in neonatology referrals at 91% compared to 45% in PCP referrals (*p* < 0.001).

In examining referrals from PED GI and PCPs, the rate of GI comorbidities was significantly higher at 56% compared to 33%, respectively (*p* = 0.023). In addition, the rate of tube feeding was significantly higher at 74% in patients referred from PED GI compared to 45% in patients referred from PCPs (*p* = 0.006).

### 3.3 External versus internal referrals comparison

The rate of internal referrals was greater at 72% of all referrals. The median age at referral from external sources was 20 months (Q1, Q3: 11, 71). In comparison, the median age at referral from internal sources was 14 months (Q1, Q3: 9, 30); however, this difference was not statistically significant (*p* = 0.059). No significant differences were noted between external and internal referrals in the rates of children who had GI comorbidities, at least four comorbidities, EGD examination, appetite‐stimulating medication use, history of tube feeding use, and use of intensive feeding therapy (Table 2). Likewise, there were no significant differences in weight *z* scores, weight‐for‐length *z* scores, and BMIs at referrals between internal and external referrals (Table 2).

**Table 2:** External and internal referral comparison of baseline characteristics of patients seen in the multidisciplinary pediatric feeding clinic.

Characteristic	*N*	External	Internal	*p* value
Referral age, median (Q1, Q3)	148	20 (11, 71)	14 (9, 30)	0.059
Female, *n* (%)	148	19 (46%)	49 (46%)	>0.9
Weight *z* score at referral, mean (*σ*)	142	−1.16 (1.53)	−1.07 (1.53)	0.8
Weight‐for‐length *z* score at referral, mean (*σ*)	100	−0.34 (1.37)	−0.39 (1.47)	0.9
BMI at referral, median (Q1, Q3)	141	16.13 (14.79, 18.50)	16.01 (14.93, 17.65)	0.6
≥1 GI Comorbidity, *n* (%)	145	13 (33%)	43 (41%)	0.4
≥4 Comorbidities, *n* (%)	146	16 (41%)	52 (49%)	0.5
History of tube feeding, *n* (%)	141	24 (63%)	66 (64%)	>0.9
Use of endoscopy, *n* (%)	144	8 (20%)	21 (20%)	>0.9
Appetite‐stimulating medication use, *n* (%)	143	6 (16%)	18 (17%)	>0.9
Use of intensive feeding therapy, *n* (%)	143	4 (10%)	11 (11%)	>0.9

Abbreviations: BMI, body mass index; GI, gastrointestinal.

### 3.4 Oral aversion and tube feeding

**Figure 2:**
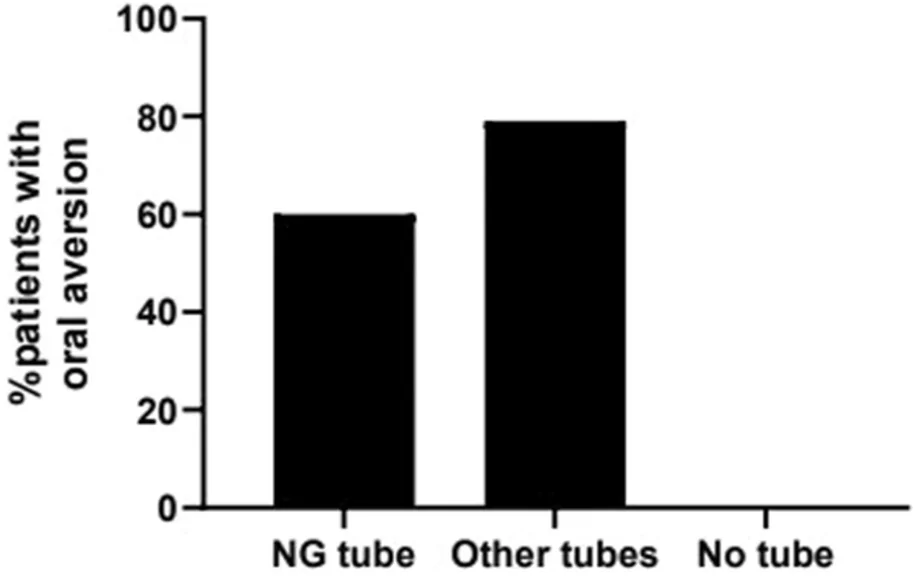
Rates of oral aversion by use and type of feeding tubes. NG, nasogastric.

We further evaluated a subset of the cohort that was representative of new patient referrals and found that 69% (45/65) had oral aversion as a listed diagnosis. This cohort was a subset of patients who were seen in our program in the 12‐month period prior to the end of the retrospective data review (Figure 2).

There was no significant difference found in the percentage of the subgroup with the diagnosis of oral aversion who only had an NG tube (60%) compared to those who had other types of tubes (79%) (*p* = 0.13). Those with other types of feeding tubes had an estimated 2.69 times the odds of oral aversion relative to those with only NG tubes. There was no reported oral aversion in patients without feeding tubes. It was also found that each 1 month increase in age at referral lowered the odds of oral aversion by 1%. However, neither of these effects was statistically significant.

## 4 DISCUSSION

Our study demonstrated that PCP referrals were the most common referral source to our program. This finding was consistent with the referral patterns observed by Sant'Anna et al. as well as Benton et al., where 60% of their patients were referred by pediatricians and 58% from PCPs, respectively.^7^, ^8^ Specialist referrals were associated with higher rates of comorbidities and tube feeding compared to PCP referrals. This pattern highlights the greater medical complexity among patients referred by specialty services. The median age of referral from external sources was 6 months greater than the median age of referral from internal sources (*p* = 0.059), despite no considerable differences in disease severity, interventions, comorbidities, or any of the other characteristics. Sharp et al. found that current treatment capacity is less than 1000 slots per year despite conservative estimates of 45,000 children under the age of 5 years who would benefit from intensive management, highlighting the dramatic unmet need among this population.^9^ Taking into consideration the high rate of referrals from PCPs, these findings highlight opportunities to improve resource utilization and disease management through the development of educational materials and outreach programs that connect feeding programs with PCPs.

We found that in the subset with oral aversion, it was noted to be a common diagnosis when a history of feeding tube use was present, regardless of the tube type, but was less prevalent in the NG tube group. More so, those with a history of other types of tubes had increased odds of oral aversion compared to the NG tube group; additionally, an increased age at referral was associated with lower odds of oral aversion. The lower rate of oral aversion in the NG group may reflect a milder severity of feeding disorder overall. Although neither of these findings was statistically significant, we hypothesized that this was likely due to the relatively small sample size. Enteral feeding has been well known to be associated with increased aversion to oral feeding^10^, ^11^; however, uncertainty has continued to exist regarding whether the method of enteral feeding affects oral aversion.^11^, ^12^, ^13^ Krom et al. noted no statistically significant differences in the rates of gagging or vomiting between patients with NG tubes and other types of tubes.^14^ These findings suggest that there may be no difference in the rates of aversive behaviors between different types of tubes used for enteral feeding, but further investigation is required to elucidate this relationship. In practice, patients and providers may be reluctant to utilize NG tube feeds with a presumed oral aversion risk. In our small cohort, tube‐fed patients were not more likely to be orally averse.

Our data found no significant differences in the rates of EGDs among the different referral sources. Currently, there is no clinical consensus or consistent recommendations regarding the use of EGDs in children with feeding disorders.^15^, ^16^ Edwards et al. reported in their retrospective study of a multidisciplinary feeding clinic that 330 patients (50.2%) had EGDs with complete biopsies.^15^ Of these patients, 40% were found to have esophagitis, 33.6% had gastritis, and 15.2% had duodenitis.^15^ In a retrospective cohort study, Robinson et al. analyzed EGD biopsy results from PFD patients compared to age‐matched controls who were referred for endoscopy for reasons other than feeding disorders.^16^ They reported that 57% of PFD patients had abnormal biopsies and 53% of control patients had abnormal biopsies.^16^ These results indicate the importance of EGDs in evaluating underlying upper gastrointestinal pathology and the need for formal recommendations for EGD in patients with PFDs.

In our clinic, cyproheptadine is the primary appetite‐stimulating medication utilized for PFDs, with 17% of the patients receiving cyproheptadine. There were no significant differences in the rates of use between the different referral sources. Similarly, there was no significant difference between the rate of use from internal (17%) and external (16%) referrals. Several studies have examined the use of cyproheptadine as an appetite stimulant for pediatric patients who are malnourished and/or underweight.^17^ In their systematic review, Harrison et al. identified 10 articles examining cyproheptadine use in malnourished and underweight children.^17^ They report that all these studies demonstrated some improvement in weight in underweight children on cyproheptadine.^17^ Thus, the use of cyproheptadine has been well established in many pediatric feeding clinics, with minimal side effects and the ability to titrate the dose and cycle dosing. However, a clear correlation with long‐term PFD resolution needs to be more closely evaluated.

Our results demonstrated that approximately 10% of patients received intensive feeding therapy. Intensive feeding therapy, and more broadly, a behavior analytic approach, has been found effective in the management of PFDs.^18^ We hypothesized that the relatively small sample size likely contributed to the rate of patients receiving this therapy; hence, it was likely underutilized. Additional variables include the need for at least some in‐person therapy, which is not always feasible for families due to work schedules and distance from our center, and our limited staffing with one therapist dedicated to feeding therapy. Limited access and the small number of slots available remain the most likely variables. Further investigation is needed to characterize intensive feeding therapy.

## 5 CONCLUSION

There has been limited literature characterizing the referral pattern for patients in multidisciplinary feeding clinics in the United States. To our knowledge, this paper is the first report of a comprehensive analysis of referral patterns to a multidisciplinary outpatient feeding disorder clinic.

This study has several limitations. The retrospective design is susceptible to documentation variability and missing data. Data were obtained from a single center, which may limit external validity. The relatively modest sample size reduced statistical power for subgroup comparisons. In addition, charted diagnoses were relied on for variables, such as oral aversion, which may vary by clinician and over time. Future efforts should focus on multi‐site, prospective studies with standardized measures as well as an increased number of patients over a longer period.

In summary, this study provides new insight into how referral patterns shape the clinical needs of patients entering a multidisciplinary pediatric feeding program and highlights actionable areas for improving care delivery. Our findings suggest that resource utilization could be improved through targeted educational initiatives for primary care providers focused on early identification of feeding concerns, optimization of initial management, and reduction of unnecessary referrals. We estimate that approximately 25% of referrals to our clinic could be deferred with enhanced education for community providers, allowing some patients to be effectively managed locally. This approach would enable our clinic to prioritize patients with greater medical complexity who are most likely to benefit from care by a multidisciplinary team. This may lead to quicker enrollment in our feeding therapy service if we have documentation that “typical” strategies are ineffective and further evaluation, as well as therapy, is needed. In addition, the creation of an accelerated referral pathway for high‐complexity patients from specialty and neonatal services may help ensure timely access to multidisciplinary care. Despite having eligibility criteria for our program, further modifications may need to be implemented, including recognizing that oral aversion is common across tube types. Incorporating standardized anticipatory guidance and earlier behavioral feeding interventions into care pathways for all tube‑fed children may help improve oral tolerance, reduce long‑term feeding difficulties, and support earlier progression toward safe oral intake. Together, these changes are intended to streamline access, allocate resources more efficiently, and enhance the overall quality of care for patients with PFDs.

## CONFLICT OF INTEREST STATEMENT

The authors declare no conflicts of interest.
